# Standardizing Depolymerization: Strategies and Performance Metrics

**DOI:** 10.1021/prechem.5c00080

**Published:** 2025-10-27

**Authors:** Céline Calvino, Diego M. Alzate-Sánchez, Jacob J. Lessard

**Affiliations:** a Cluster of Excellence livMatS, University of Freiburg, Freiburg 79110, Germany; b Department of Chemistry and Chemical Biology, 12434 Northeastern University, Boston, Massachusetts 02115, United States of America; c Department of Chemistry, University of Utah, Salt Lake City, Utah 84112, United States of America

**Keywords:** depolymerization, self-immolative polymers, polymer recycling, stimuli, standardization, potential energy surface, closed-loop systems

## Abstract

The widespread use of polymeric materials has brought unparalleled convenience and utility, but their environmental persistence presents a critical and growing challenge. As demand increases for sustainable solutions to polymer waste, depolymerization continues to be a promising strategy for achieving true circularity. In this Perspective, we examine depolymerization from a fundamental standpoint, aiming to rationalize the advantages, limitations, and future directions of state-of-the-art technologies. We advocate for standardized reporting practices to enable meaningful comparisons across studies and, in alignment with this goal, we provide key metrics and contextual information throughout the article to support consistent evaluation of different depolymerization strategies. Ultimately, we hope to inspire readers to explore innovative and scalable solutions that advance the transformative potential of depolymerization toward the realization of a circular polymer economy.

## Graphical abstract

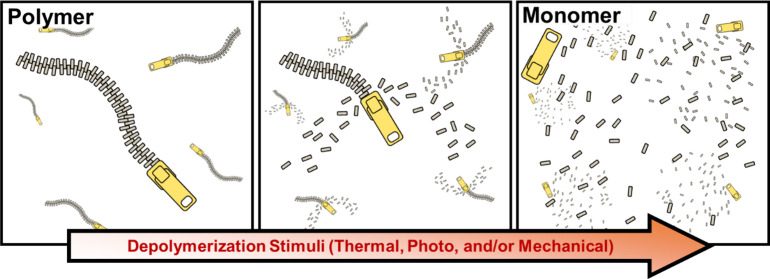

## 1 Introduction

The lightweight nature, cost-effectiveness, and extensive durability of macromolecular materials are integral to modern life. However, effective solutions for their environmental consequences have yet to meet their growing demand.^1^ The widespread use of polymer materials has led to significant challenges, including pollution and the accumulation of plastic waste, posing risks to ecosystems and human health.^2–4^ This pressing issue has propelled the fields of sustainable polymer production, (re)­functionalization, deconstruction, and closed-loop reprocessing to the forefront of research, prompting efforts to develop innovative approaches to address these environmental impacts.^5–7^

Various methods to revitalize polymer waste are being actively explored, which can be categorized into three main strategies based on their final product form: down-converting (or downcycling, which generates materials of lesser value), recycling (which restores the original material for reuse), and up-converting (or upcycling, which produces higher-value materials that can serve new purposes). Each of these strategies plays a crucial role in promoting sustainability and reducing reliance on virgin materials and their petrochemical feedstocks. Among them, recycling stands out as the only true circular process, allowing for multiple turnovers and facilitating the continual reuse of materials.

Recycling can be further divided into three methods: reprocessing, disassembly, and depolymerization (Figure 1). Reprocessing, which involves the direct recycling of polymer materials using straightforward stimuli (e.g., heat and compression), is the cornerstone of current recycling strategies.^8–10^ For example, the bulk rearrangement (reshaping) of thermoplastics or reprocessable thermosets in their molten/rubbery state is a ubiquitous approach for repurposing polymeric materials at their end-of-life stage.^11–19^ Despite being the most straightforward approach, the mixed nature of plastic waste complicates direct reprocessing, often leading to a reduction in the mechanical properties of the reprocessed materials due to the presence of various impurities (i.e., other polymers and plasticizers) or degradation (e.g., chain scission or oxidation).^20^

**Figure 1:**
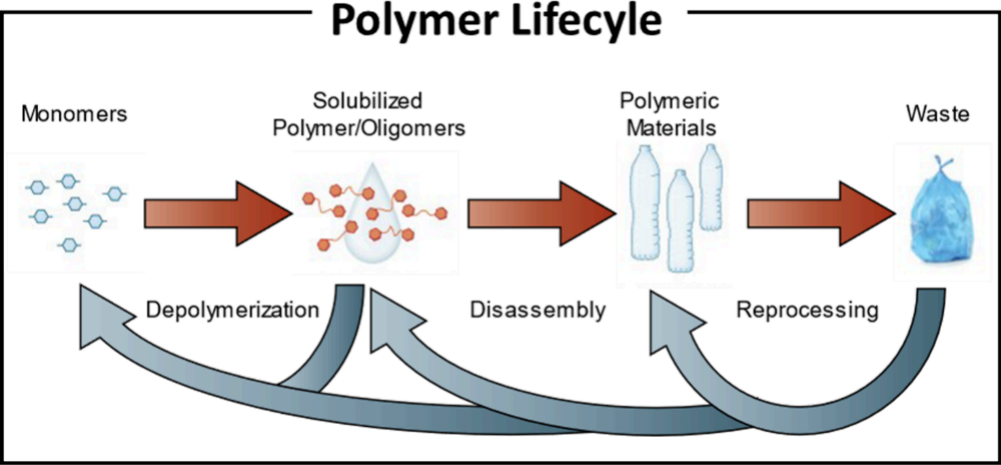
Traditional linear polymer life timeline with general methods of achieving material circularity (reprocessing, disassembly, and depolymerization).

The second method, disassembly, entails dissolving bulk materials or waste either directly or via chemical deconstruction processes (i.e., chemical recycling). For example, solvent-assisted breaking of reversible cross-links in dynamic polymer networks followed by purification enables the reprocessing of their soluble monomer/oligomer fragments.^21^ This approach allows mixed polymer waste to be separated based on solubility differences or through polymer purification. However, because most plastic waste comprises hydrocarbon backbones, separating different polymers becomes increasingly challenging due to issues like poor solubility, additives, and similar polarities.^22^ Given the complexities of both reprocessing and disassembly—particularly in reconciling mixed or degraded plastic waste—depolymerization offers a fundamentally different approach by converting polymers back to their constituent monomers. This molecular-level recycling enables true circularity, albeit through more chemically complex routes. Recent innovations in stimuli-responsive materials and catalysis have brought the practical utility of depolymerization to the forefront, making this a timely moment to evaluate its progress, challenges, and transformative potential.^23–27^ While reprocessing and disassembly remain important in current recycling infrastructures, their effectiveness is constrained by feedstock purity, material compatibility, and degradation of properties. In contrast, depolymerization can enable high-value feedstock recovery with the immediate potential for closed-loop reuse. However, the diversity of depolymerization strategies and the lack of standardized reporting has made it difficult to compare outcomes or assess reproducibility across this emerging field. Standardized practices and metrics are essential for advancing the field of polymer recycling, particularly in emerging strategies such as depolymerization. Without consistent reporting, it is difficult to compare results across studies, evaluate reproducibility, or identify the most effective approaches. Clear standards, covering not only reaction conditions but also outcomes such as monomer recovery yields, purity, selectivity, and byproduct characterization, provide a common framework that ensures transparency and comparability. These practices accelerate progress by enabling researchers to build upon one another’s work, minimize redundancy, and better assess the scalability and industrial potential of new technologies. Ultimately, standardized reporting is a critical step toward translating laboratory advances into reliable, impactful solutions that can meaningfully address the growing challenge of plastic waste.

In this Perspective, we examine depolymerization as an emerging strategy for polymer recycling and advocate for standardized reporting practices to enable meaningful comparison across methodologies. In addition to reaction conditions, we argue that monomer recovery yield and purity/selectivity (spectroscopic purity and byproduct characterization, repolymerization characterization, etc.) should also be routinely reported. These and other relevant variables—when disclosed—are summarized in Table 1 at the end of the manuscript. Our discussion is organized by nature of the applied stimuli (specifically, thermal, photo, and mechanical), presenting the current state-of-the-art while identifying areas for improvement. We critically assess challenges in reproducibility and include selected case studies to illustrate both progress and persistent limitations. Our objective is to equip both new and experienced researchers with a comprehensive understanding of depolymerization and its transformative potential in advancing recycling efforts, especially for polymers.

## 2 Thermodynamics and Kinetics of Bulk Thermal Depolymerization

To begin our discussion on thermal depolymerization, which is arguably the most commonly employed stimulus, we believe the best starting point is examining the thermodynamics of the underlying process. Achieving efficient polymerization requires sufficient enthalpic favorability to outweigh the negative entropic penalty associated with each added monomer unit. When polymerization is highly favorable, the reaction can proceed at relatively low temperatures due to a modest activation barrier (Figure 2A). However, because polymerization is thermodynamically favored, depolymerization requires overcoming a substantial activation energy, typically requiring high temperatures that can lead to undesirable side reactions (Figure 2B).^26^

**Figure 2:**
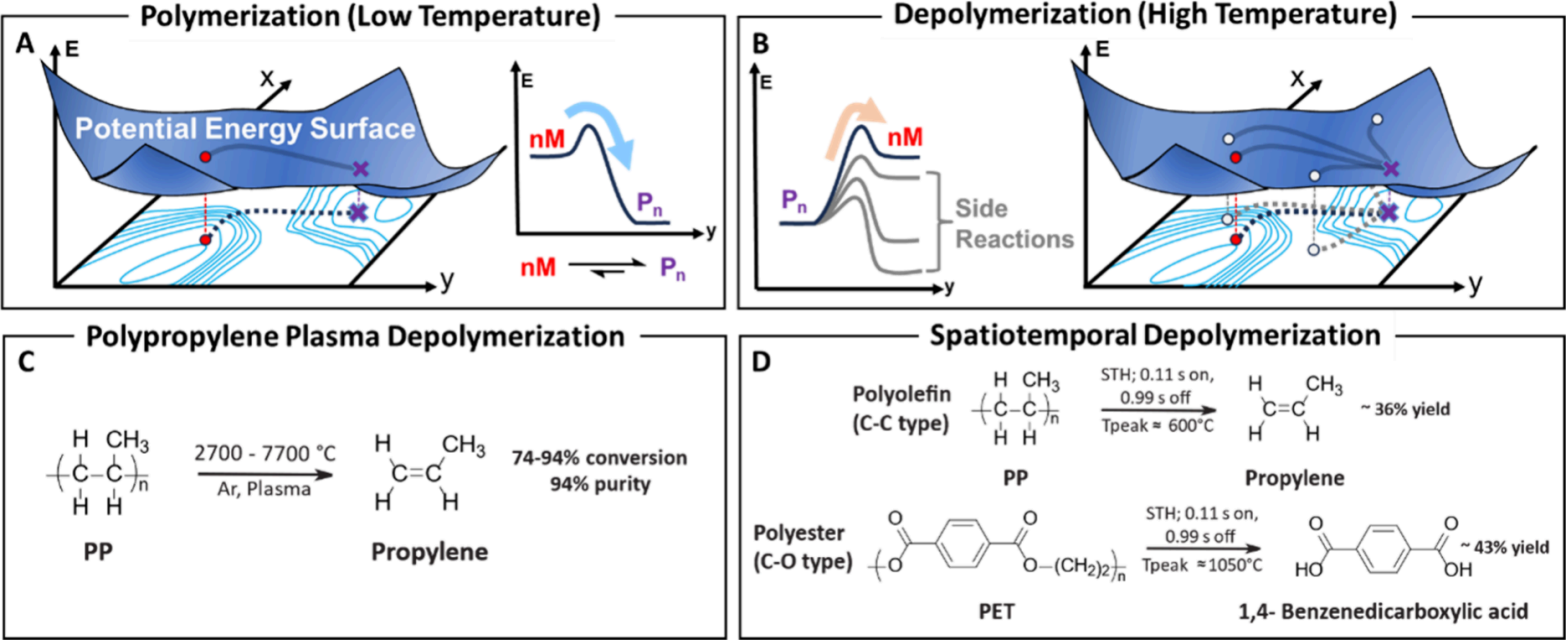
Hypothetical potential energy surface for (A) polymerization and (B) depolymerization. (C) Reaction scheme for polypropylene depolymerization through rapid heating from an induction-coupled plasma reactor from ref 33. (D) Reaction scheme for polypropylene and polyethylene terephthalate depolymerization from electrified spatiotemporal heating from ref 34.

For chain-growth polymers composed of exclusively hydrocarbon backbones, this overview may oversimplify the complexities involved. In chain-growth polymerization, propagating chain ends are often irreversibly terminated, requiring the formation of new active groups during thermolysis (heating materials until thermal decomposition). These active groups—specifically the radicals generated from homolytic bond cleavage—require additional energy to activate, which can further exacerbate the occurrence of deleterious side reactions. A notable example is the depolymerization of polyethylene (PE)—the most abundant type of polymer waste—typically conducted through pyrolysis. During this process, the radical homolytic bond cleavage has been shown to produce a variety of alkenes and alkanes of variable lengths in a nonselective manner.^28^ Despite recent advancements in pyrolysis techniques, the current state-of-the-art in PE depolymerization yields a monomer recovery of only about 22–27% ethylene (Table 1, entry 1).^29^

The predilection toward inimical reactions resulting from thermal depolymerization is further demonstrated by polypropylene (PP) deconstruction, the second most widely used commodity polymer. During traditional thermolysis (under N_2_), PP typically produces only ∼10 wt% of the original monomer (Table 1, entry 2).^30^ Although PP degradation proceeds via a more stable secondary radical chain end, unlike polyethylene (PE) that forms less stable primary radicals, this advantage does not inherently translate to efficient depolymerization. Even when catalytic strategies are employed to lower the activation energy and reshape the potential energy surface in favor of monomer recovery, yields remain low.^31,32^

Beyond thermodynamic considerations, the yields from thermolysis can also be influenced by the kinetics of depolymerization. Kinetics of depolymerization generally rely on the rate of heating, or how rapidly the activation energy barrier can be overcome such that side reactions are outcompeted. In 2000, Knight and co-workers demonstrated how kinetics can drive selective thermal depolymerization of PP using plasma.^33^ In their study, an induction-coupled plasma (ICP) reactor was employed under argon, heating PP to temperatures between 2700 and 7700 °C, with a heating rate of 10^6^ °C/s and a cooling rate of 1000 °C/s. These extraordinarily high temperatures and extreme heating rates enabled the depolymerization of PP, resulting in an average of 78% conversion to gaseous products with 94% propylene purity (Figure 2C and Table 1, entry 3).

This concept of rapid heating rates on pyrolysis efficiency was also recently demonstrated by Ju, Hu, and co-workers for the depolymerization of both PP and polyethylene terephthalate (PET).^34^ The team used electrified spatiotemporal heating (STH) with a bilayer structure of porous carbon felt, in which the top electrically heated layer generates a heat gradient promoting continuous melting, wicking, and vaporization to achieve localized heating to pyrolysis temperatures within a fraction of a second. With pulse cycles of 0.11 s on and 0.99 s off, PP and PET were heated to 600 and 1100 °C, respectively, in a rapid and spatioselective manner. These separate reactions yielded 36% propylene from PP and 43% 1,4-benzenedicarboxylic acid from PET, without the use of catalyst (Figure 2D and Table 1, entries 4 and 5). The authors noted that the monomer yield could be further optimized by altering the material properties of the reactor layer, such as pore shape, pore size and distribution, and surface energy. Importantly, both examples highlight the significant kinetic contribution of heating rates to the efficiency of the depolymerization process through the circumvention of undesirable side reaction.

### 2.1 Bulk Thermal Depolymerization of Polymers with “Weak Links”

Understanding the interplay between kinetic and thermodynamic contributions to depolymerization presents opportunities for enhancing the effectiveness of polymer deconstruction strategies. For instance, decreasing the energy barrier to active group formation by installing “weak links” (thermally labile bonds) could facilitate depolymerization under milder conditions. This approach enables the formation of active groups at significantly lower temperatures than those employed in traditional bulk thermolysis, resulting in a more controlled and efficient unzipping process.

Depolymerization of hydrocarbon-based polymers, which are prevalent in most commodity materials, can be initiated by uncapping labile end groups, cleaving labile pendent groups, or through direct chain scission of the polymer backbone. All these methods create active sites (e.g., radicals) on the polymer chain that promote depolymerization at lower temperatures due to being under nonequilibrium conditions (i.e., vacuum to remove monomers), thereby reducing the risk of unwanted high-temperature side reactions.^35^

A noteworthy example of uncapping labile end groups is provided by Anastasaki and colleagues, who explored the bulk depolymerization of polymethylmethacrylate (PMMA) made from reversible addition-fragmentation chain-transfer (RAFT) polymerization with dithiobenzoate end groups and atom transfer radical polymerization (ATRP) with bromide end groups (Figure 3 and Table 1, entry 6).^36^ Their study demonstrated that generating an alkene end group—through the *in situ* elimination of either the dithiobenzoate or bromine—greatly enhanced the rate of bulk depolymerization, achieving an impressive yield of 84% monomer. This innovative strategy highlights the potential of using end groups to create active sites that facilitate depolymerization at lower temperatures, ultimately improving the efficiency of the process.

**Figure 3:**
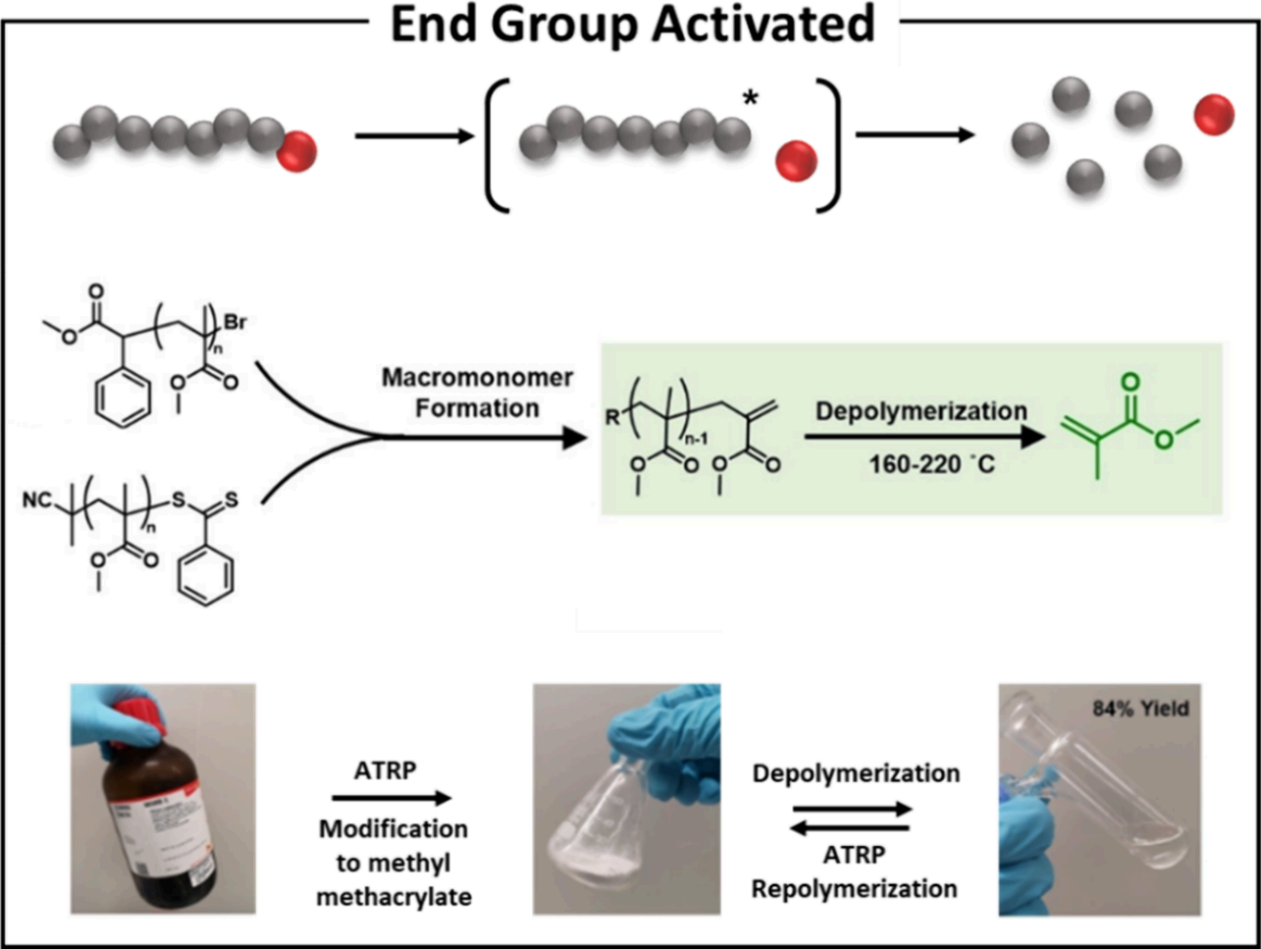
End-group activated radical depolymerization from pyrolysis of poly­(methyl methacrylate) derived from controlled radical polymerization. Reproduced from ref 36. Licensed by CC-BY 4.0 2023, Wiley-VCH GmbH, Angew. Chem. Int. Ed.

Like end-group activation, pendent groups can also be utilized for the depolymerization of hydrocarbon backbones. It was recently demonstrated by Sumerlin et al. that the addition of a weak link as a pendent group can be effective for the depolymerization of methacrylic backbones (Figure 4A and Table 1, entry 7).^37,38^ Methyl methacrylate was copolymerized with a methacrylate containing phthalimide pendent groups to form both linear polymers and networks. Thermal gravimetric analysis (TGA) of the copolymers indicated a significant reduction of the onset temperature of depolymerization (the temperature at which 5% mass loss is achieved) through monomer volatilization, from over 300 to 180–230 °C with only 1–5 mol% loading of the phthalimide comonomer. Mechanistically, upon heating, the phthalimide is fragmented homolytically into a persistent radical, releasing an equivalent of carbon dioxide and leaving a backbone-centered radical, which initiates depolymerization through β-scission. It was further demonstrated by the authors that this method could be applied to both bulk linear polymers and cross-linked materials, achieving high monomer yields (up to 85% depolymerization) compared to pure PMMA, which showed only up to 50% depolymerization. This innovative use of a destructive pendent group exemplifies a strategy for transferring the active group from the side chain to the primary chain, effectively activating depolymerization with minimal incorporation of bespoke monomer.

**Figure 4:**
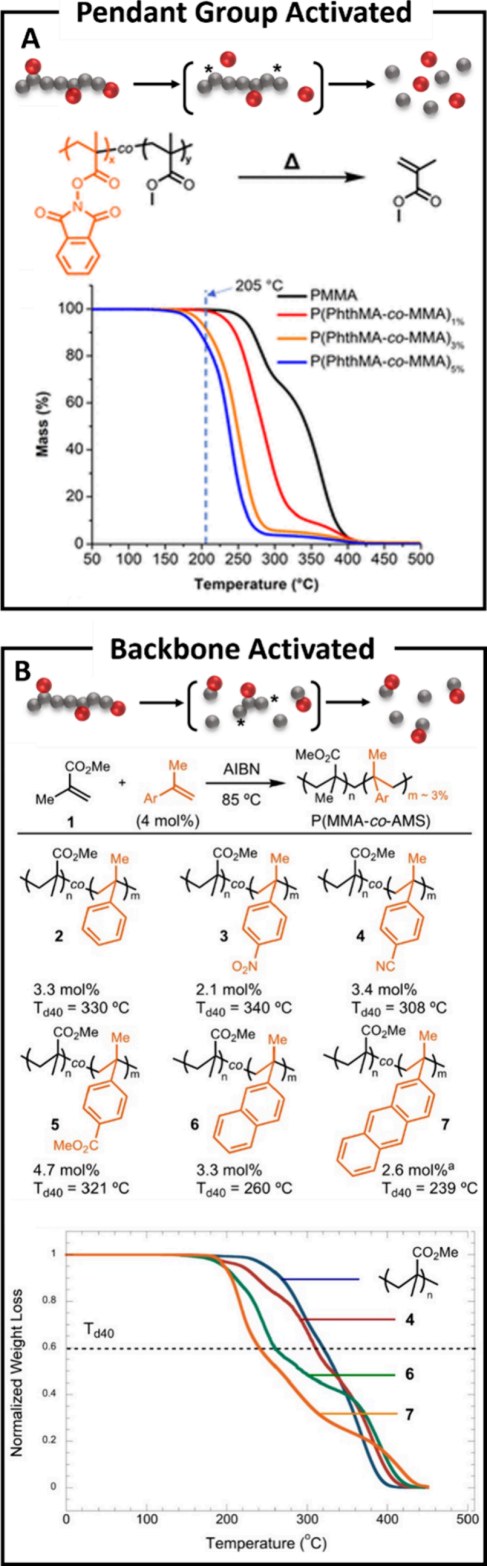
(A) Poly­(methyl methacrylate) (PMMA) depolymerization from pendant phthalimide “weak links” for radical activation. TGA shows a reduction in the onset temperature for depolymerization compared to unfunctionalized polymer. (B) PMMA doped with α-methylstyrene-type (AMS) monomers for backbone “weak links” depolymerization. TGA shows reduction in thermolysis temperature with various AMS monomers. Reproduced from (A) ref 37 and (B) ref 39. Copyright 2024 American Chemical Society.

Concurrently, Liu and co-workers demonstrated a similar strategy using PMMA doped with α-methyl styrene (AMS) derivatives as comonomers (Figure 4B and Table 1, entry 8).^39^ Unlike the decomposition of phthalimide, which leads to an active group on the backbone, the authors propose that the low ceiling temperature of the AMS monomer undergoes direct homolytic bond cleavage at the comonomer, subsequently leading to depolymerization. To enhance bulk depolymerization efficiency, the electronic properties of the AMS monomer were modified to further delocalize and stabilize the formed radical, thereby lowering the activation barrier of homolytic bond scission. The studies revealed that the most effective comonomer was an anthracene pendent group, which exhibited 60% mass loss (*T*_60%_) at 260 °C, compared to α-methyl styrene, which had a *T*_60%_ at 330 °C. The anthracene-functionalized AMS copolymer was heated at 180 °C for 6–8 h under neat conditions, yielding 78% MMA recovery with only 3 mol% AMS loading. To further illustrate the weakening of the backbone, the authors calculated the bond dissociation energy of a head-to-tail MMA-MMA linkage, finding it to be 77.2 kcal/mol (323 kJ/mol). In comparison, the MMA-AMS anthracene linkage (Figure 4B, molecule 7) showed a reduced bond dissociation energy of 71.3 kcal/mol (298 kJ/mol), indicating a more thermolytically labile bond. Overall, this strategy highlights the effectiveness of using weakened backbone linkages to achieve polymer deconstruction at lower temperatures.

All three strategies mentioned above required monomer functionalization at the beginning of the polymerization process, which excludes them from recycling current PMMA waste. Building on their earlier work, Sumerlin and co-workers sonochemically fragmented PMMA *in situ* in the presence of phthalimide methacrylate (0.25–1 mol%), resulting in chain extension of the polymer fragments, thereby introducing weak links.^40^ The resulting PMMA-*b*-poly­(phthalimide methacrylate) was then heated at 290 °C for over an hour, resulting in an 81% mass loss within the first 30 min (Table 1, entry 9). While the yield of recovered monomer was reported for the PMMA system, the similar depolymerization efficiency observed in previous studies supports this outcome. The authors also fragmented poly­(α-methyl styrene), a polymer known for its low ceiling temperature (61 °C), through sonication to chain extend with phthalimide methacrylate. This modification enabled depolymerization at 220 °C, resulting in a 70% monomer recovery of α-methyl styrene monomer. This work underscores the potential of using postpolymerization modifications to enhance depolymerization efficiency (Table 1, entry 10).

In addition to modifying existing commercial polymer materials or creating modified analogs, novel systems have been developed that target low depolymerization temperatures in bulk (Table 1, entry 11).^41–43^ These methods often emphasize sourcing monomers from biosourced reactants for polymer materials, thereby bypassing traditional petrochemical feedstocks. These approaches focus on lowering activation barriers to monomer as *vide supra* but with predesigned weak linkages. While these approaches are innovative, they require further investigation to evaluate their industrial relevance and likely will encounter significant activation barriers to implementation.

In summary, while thermal depolymerization of commodity polymers faces substantial challenges due to unfavorable thermodynamics, high activation barriers, and competing side reactions, recent advances demonstrate that both kinetic control and strategic structural modifications can significantly improve depolymerization efficiency. Rapid heating techniques and the incorporation of thermally labile “weak links”—whether at end groups, pendent positions, or along the backbone—offer promising avenues to lower operational temperatures and enhance monomer recovery. These strategies not only deepen our mechanistic understanding of depolymerization but also lay the groundwork for designing polymers with end-of-life recyclability in mind.

## 3 Photodriven Depolymerization

Photochemical depolymerization offers a compelling alternative to traditional thermal methods, enabling selective bond cleavage with significantly lower energy input. Unlike thermolysis—which requires energy inputs exceeding 2 kJ/g^44^ and often leads to undesired side reactions that reduce monomer purity—photoinduced processes operate under mild conditions, thereby reducing byproduct formation and preserving the chemical integrity of recovered building blocks. By selectively exciting molecules to higher electronic states, photoactivation can modify the reaction potential energy surface (PES), opening energetically favorable pathways for bond scission (Figure 5).

**Figure 5:**
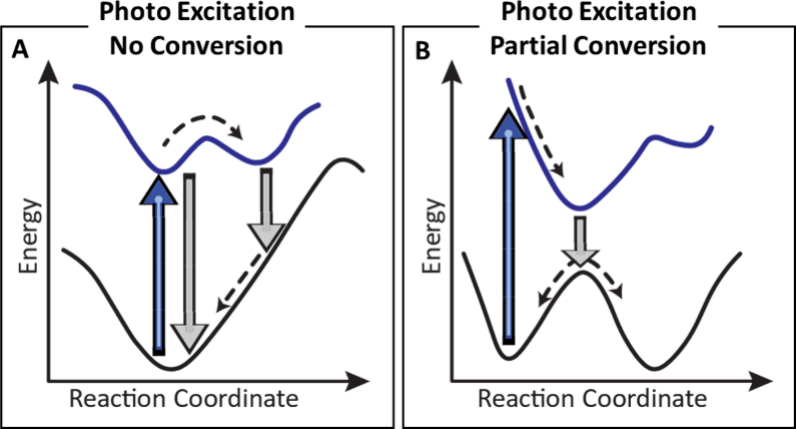
Schematic energy profiles of the ground state and first excited state for two systems: (A) one that undergoes an excited-state reaction but returns to the ground state without chemical conversion and (B) one that achieves partial chemical conversion upon relaxation to the ground state. Blue arrows indicate light absorption, while gray arrows represent light emission.^49^

Figure 5 illustrates two simplified potential energy diagrams contrasting photochemical and thermal reaction pathways.^49^ In Figure 5A, the molecule absorbs light and is excited to a higher electronic state but subsequently returns to its original ground-state geometry without undergoing any chemical transformation. Conversely, Figure 5B depicts a productive photochemical pathway where excitation leads to a conformational rearrangement, resulting in a new minimum on the excited-state potential energy surface. Relaxation back to the ground state occurs at a high-energy geometry that circumvents the significant activation barrier typically required in thermal processes, facilitating chemical transformation. This mechanism accounts for the enhanced efficiency of photochemical activation compared to thermal activation, as it selectively targets light-absorbing molecules, thereby reducing energy waste and minimizing side reactions. The likelihood of a productive photochemical event depends critically on the topology of the potential energy surfaces and the nuclear dynamics governing the excited state. Together, these scenarios emphasize the unique ability of photochemistry to exert precise, energy-efficient control over molecular reactivity beyond the reach of conventional thermal methods.

A key advantage of photodriven strategies lies in their spatiotemporal control.^45–47^ Reaction parameters such as wavelength, light intensity, and irradiation duration are tunable, allowing tailored activation of specific chemical motifs.^48^ This level of control is especially valuable for advancing sustainable recycling technologies by enabling selective depolymerization of target polymers while minimizing collateral degradation. In industrial settings, ultraviolet (UV) irradiation is widely used to induce photodegradation of plastics in bulk, often leading to polymer embrittlement, discoloration, and fragmentation.^50,51^ However, such bulk degradation tends to be a time-consuming process and typically yields low-value fragments rather than recoverable monomers, limiting its utility for circular recycling.^52^ To improve effectiveness, photodegradation is often combined with thermal or chemical processes, though these hybrid approaches still lack molecular precision. Building on this premise, a critical challenge in the field is to leverage the unique attributes of light for precise polymer deconstruction into their original building blocks rather than fragmentary breakdown. To this end, several recent advances focus on integrating photochemical activation with thermally assisted depolymerization in engineered systems—particularly those incorporating “weak links” or labile end groups designed to initiate head-to-tail disassembly.

### 3.1 Photothermal Depolymerization of Polymers with “Weak Links”

As previously described, polymers designed to depolymerize through a head-to-tail unzipping mechanism represent a compelling route for controlled chemical recycling. These systems rely on incorporating ″weak links″—structural features such as cleavable end groups or low-stability bonds—that, once activated, initiate sequential monomer release without random chain scission. Photothermal strategies can enable spatiotemporal control over this process. In particular, light-triggered removal of stabilizing end groups exposes a reactive chain end that triggers depolymerization in a domino-like fashion. This approach offers high monomer recovery, avoids random bond breaking, and reduces unwanted byproducts.^53–55^

#### 3.1.1 Photoinduced Depolymerization of Polymers with Reactive Pendent Groups

Yaguchi and Sasaki developed poly­(olefin sulfone) bearing photoresponsive pendent groups, including *o*-acyl oxime, 3,5-dimethoxybenzyloxy carbonyl, oxime urethane, and *o*-nitrobenzyl)­oxy]­carbonyl. Upon UV irradiation (254–365 nm), these groups cleave, releasing amines that catalyze the depolymerization of the polymer backbone into olefins and sulfur dioxide (Figure 6 **A**).^56^ The team demonstrated that irradiating a 1 mm-thick polysulfone film at 600 mJ/cm^2^, followed by mild heating at 150 °C for 15 min, resulted in 95% degradation and approximately 50% monomer recovery (Table 1, entry 12). Importantly, the efficiency of depolymerization was found to correlate with the strength of the released photobase. Furthermore, while sensitizers such as benzophenone have shown to accelerate the reaction rate, their utility came at the cost of generating nonrecyclable residues, which compromised the overall circularity of the system. Despite this drawback, this work laid the foundation for phototriggered depolymerization strategies, which have since been adapted for applications including dendrimer degradation,^57^ block copolymers, and polymer nanoparticles,^58^ in drug delivery systems^59^ and in the development of transient and self-destructive devices.^60^

**Figure 6:**
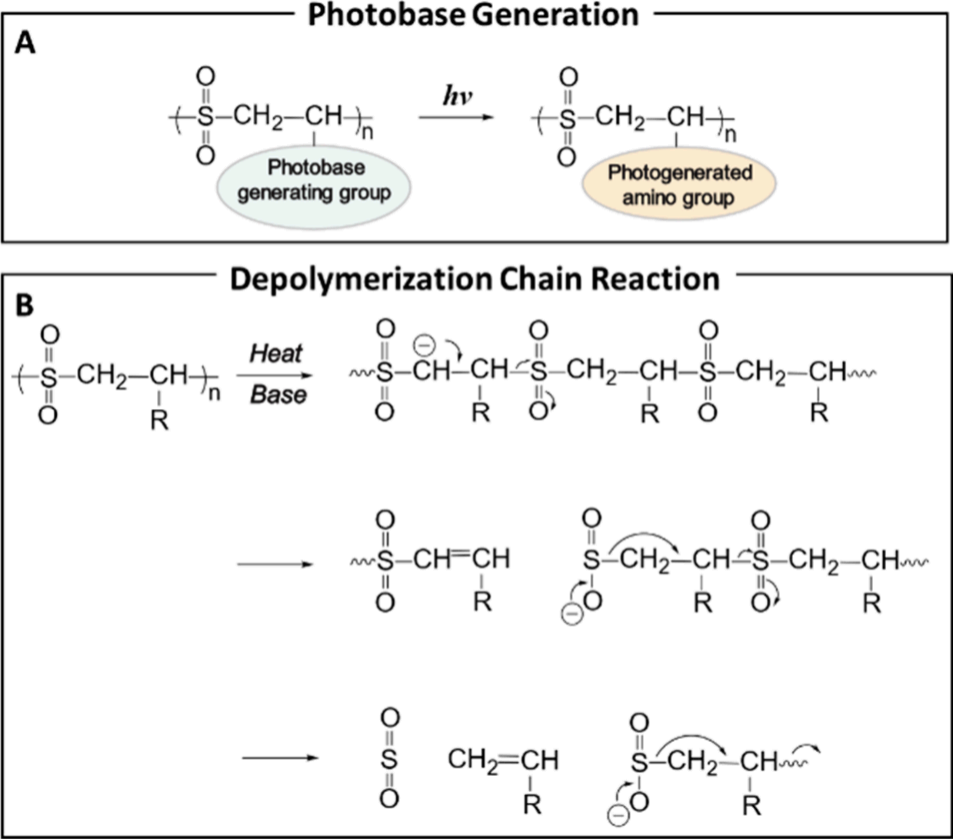
(A) Photoinduced depolymerization of 1:1 alternating poly­(olefin sulfone)­s containing photobase generating groups in the side chain. (B) Proposed mechanism of base-catalyzed thermal depolymerization of 1:1 alternating poly­(olefin sulfone)­s. Reproduced from ref 56. Copyright 2007 American Chemical Society.

However, translating these promising results to solid-state systems remains challenging. Limited molecular mobility in bulk materials hinders efficient photoinduced depolymerization, which has led to a decline in research interest in this area compared to solution-phase systems. Designing photocleavable groups that directly trigger depolymerization cascades is complex and must also ensure compatibility with various polymer matrices and processing conditions. Most current systems rely on reactive intermediates requiring additional chemical steps, limiting their applicability in bulk materials.^55^ In contrast, approaches coupling photochemical activation with thermal input show promise in overcoming these barriers, enabling more straightforward degradation pathways suited for recyclable bulk polymers.

#### 3.1.2 Selective Radical Photo-Thermal End-Group Chain Depolymerization

RAFT and ATRP-derived polymers, especially PMMA, have served as powerful platforms to investigate light-activated depolymerization via end-group reactivity. In a seminal study, Sumerlin and colleagues investigated the photolysis of C–S bonds in thiocarbonylthio-capped PMMA (6000 Da) prepared via RAFT polymerization. They demonstrated depolymerization via a photoiniferter mechanism, enabling precise control over polymer chain length (Figure 7 **A**).^61^ Specifically, irradiating PMMA (5 mM in dioxane at 100 °C) with green (515 nm) and UV (365 nm) light significantly enhanced depolymerization rates compared to thermal processes alone. This effect is attributed to the accelerated homolytic cleavage of the terminal C–S bonds, increasing the concentration of active radicals and promoting efficient unzipping. As a result, depolymerization reached 87% within 1 h, substantially higher than the 70 ± 0.5% observed after 24 h under thermal conditions alone (Table 1, entry 13). The team further noted a strong dependence on irradiation wavelength, with UV light accelerating depolymerization of dithiobenzoate end groups and blue light enabling complete depolymerization of trithiocarbonate (TTC) end groups within 24 h. Notably, these experiments were performed under dilute conditions to effectively lower the ceiling temperature, thereby shifting the thermodynamic equilibrium toward depolymerization and enabling more efficient monomer recovery. These findings highlight the critical role of both wavelength and concentration in maximizing photoreactivity.

**Figure 7:**
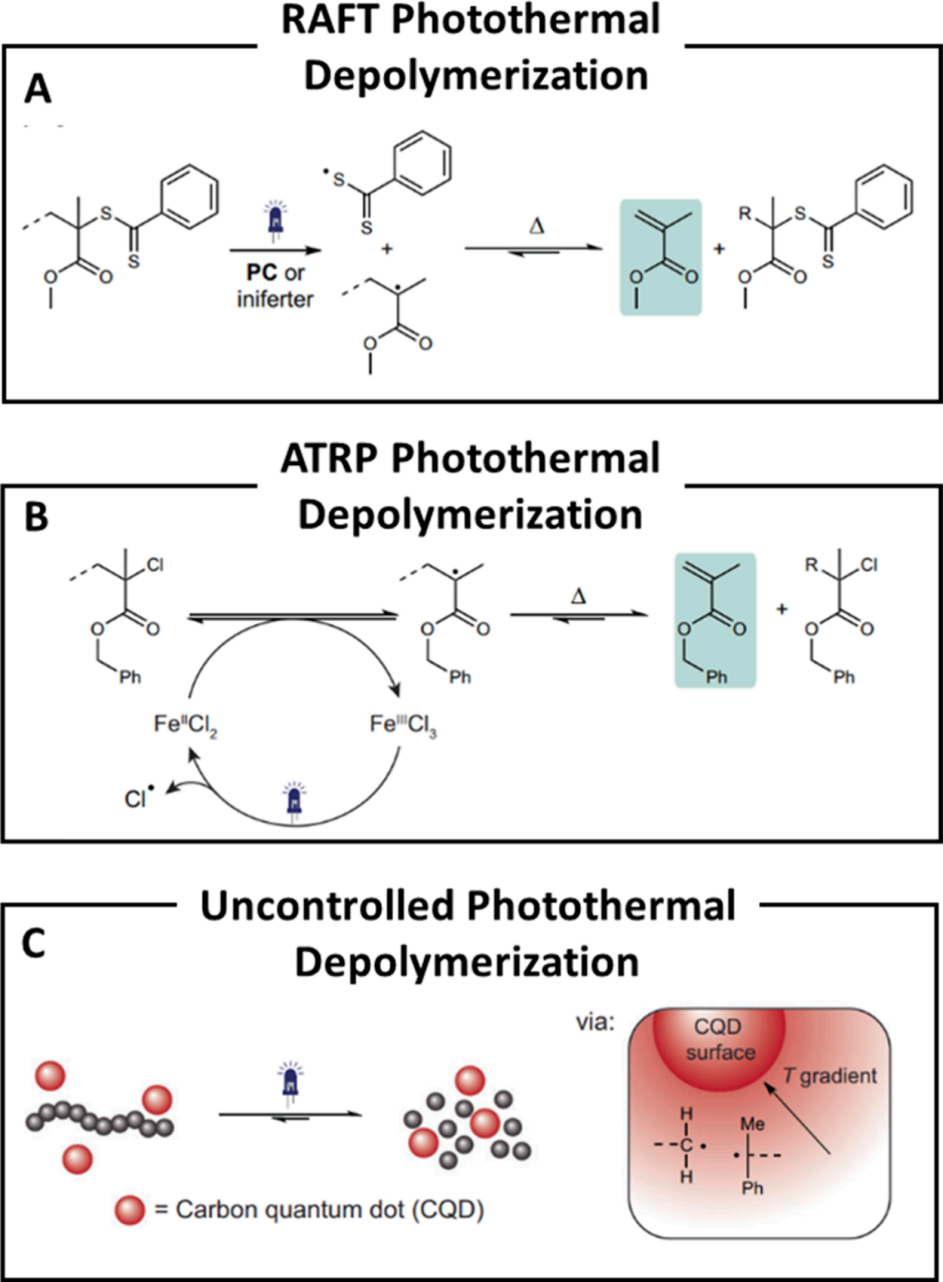
(A) Controlled photothermal depolymerization by reversible addition-fragmentation chain-transfer (RAFT) photoiniferter or photocatalytic (PC) processes. (B) Controlled photothermal depolymerization by atom transfer radical polymerization (ATRP) mechanisms. (C) Schematic of uncontrolled photoinduced thermal depolymerization in presence of carbon quantum dots. Reproduced with permission from ref 47. Copyright 2025 Nature.

In parallel, Anastasaki and co-workers reported light-accelerated depolymerization of RAFT-synthesized PMMA catalyzed by eosin Y through end-group activation.^62^ In their system, PMMA (Mn ∼6300 Da) underwent depolymerization to yield up to 82% monomer within 8 h (Table 1, entry 14), using only 100 ppm eosin Y, green light (2.31 mW cm^–2^), and mild heating (100 °C, 5 mM in dioxane). Importantly, this method proved compatible with various light sources (e.g., blue, red, and white light irradiation), solvents, and RAFT agents, showcasing its robustness and versatility. It is worth mentioning that although light was used to initiate the depolymerization, thermal effects continued to sustain the reaction even after irradiation ceased—underscoring the synergistic nature of photothermal activation.

In a joint effort, Anastasaki and Matyjaszewski’s teams applied a similar strategy to depolymerize poly­(benzyl methacrylate) (PBzMA) via photoinduced ATRP using ppm-level concentrations of iron-based catalysts (Figure 7B). In this system, FeCl_2_ activates the polymer chain ends through oxidation to FeCl_3_ upon light exposure, triggering controlled depolymerization. This process significantly reduced the depolymerization temperature from 170 to 100 °C and allowed precise temporal control simply by switching the light source on or off. Moreover, it supported high polymer loadings (up to 2 M) and enabled a monomer recovery of up to 90% (Table 1, entry 15). The method was further shown to be compatible with various monomers and light sources, emphasizing its adaptability.^63^

Although photothermal depolymerization strategies offer significant advantages over purely thermal methods—such as lower operating temperatures, wavelength tunability, and improved control—their reliance on solvents, specific catalysts, and oxygen-free environments limits large-scale implementation. Extending these methods to bulk materials requires careful adaptation to maintain efficiency while enhancing scalability and practicality. This challenge partly arises from the way light interacts with the polymer: Upon photon absorption, the polymer is promoted to an excited electronic state, effectively reshaping the potential energy surface by enabling reaction pathways that are inaccessible thermally. While this concept helps explain the unique photochemical activation, it is somewhat abstract and highlights the need for tailored photochemistry design to make depolymerization more viable in bulk systems.

### 3.2 Non-Selective Photoinduced Thermal Depolymerization

Bulk depolymerization often suffers from uncontrolled side reactions, particularly oxidative degradation arising from reactive intermediates generated under intense light and/or high temperatures, which reduces monomer yield and complicates purification. To address this, the Stache group developed a photothermal recycling strategy using carbon quantum dots (CQDs) in the presence of ZnO or BaO catalysts. Upon broad-spectrum light exposure, CQDs convert absorbed photons into localized heat, reaching temperatures above 6000 K at the nanoscale, while maintaining the bulk matrix below 290 °C. These extreme, transient thermal spikes enable selective C–C bond cleavage without bulk overheating. Combined with vacuum distillation, this photothermal method enabled efficient depolymerization of poly­(α-methyl styrene) (PAMS), polyphthalaldehyde (PPA), polystyrene (PS), polylactic acid (PLA), and PMMA, achieving monomer yields of 43–95% (Figure 7C). Moreover, compared to conventional bulk heating, oxidized byproducts were reduced from 16 to 1%.^64^ As in rapid thermal depolymerization, maintaining low concentrations of reactive intermediates helps suppress side reactions. Additionally, localized heating generated by the CQDs promotes efficient monomer diffusion away from hot zones, further reducing degradation. Notably, the spatial control offered by CQDs enables selective depolymerization within polymer mixtures. By embedding CQDs only in specific components—typically those with lower decomposition temperatures—selective recycling can be achieved. This approach has been successfully demonstrated in PAMS–PMMA and PAMS–PS blends, yielding high conversion and excellent monomer purity (Table 1, entry 16). Overall, this quantum dot-assisted strategy stands out as one of the most promising photodriven depolymerization methods, enabling selective, energy-efficient monomer recovery with high purity. By lowering processing temperatures—often exceeding 300 °C for commodity plastics—it offers a compelling alternative to pyrolysis and mechanical recycling. However, to fully realize its potential for sustainable, large-scale polymer recovery, scaling beyond gram-level demonstrations and improving spatiotemporal control are essential.

### 3.3 Wavelength Covalent-Gated Ligation

To fully exploit the spatiotemporal control afforded by photoreactions, wavelength-gated covalent chemistries have emerged as a promising approach for reversible polymerization.^65,66^ These frameworks employ photoswitchable molecules capable of forming and cleaving covalent bonds in response to specific wavelengths of light, enabling photoinitiated polymerization and depolymerization of monomeric building blocks.^67^

Currently, only a limited number of reaction pathways exhibit reversibility when stimulated by light. A prominent example is the reversible [2π + 2π] photocycloaddition, where distinct wavelengths trigger cycloaddition and cycloreversion processes.^68^ This light-mediated reaction involves excitation of conjugated alkenes to form cyclobutane rings within specific wavelength ranges. These cyclobutanes can be reverted to their original alkenes upon exposure to light at lower wavelengths, providing an on-demand orthogonal process and facilitating iterative cycles of reversible reactions. However, only a few chemical structures, including cinnamic acid,^69^ thymine,^70^ coumarin,^71^ and stilbene,^72^ have been identified to undergo these reversible light-mediated [2π + 2π] cycloadditions (Figure 8A). In reversible polymerization, these motifs enable light-triggered cross-linking or chain extension, while typically permitting only partial deconstruction rather than full recovery of the original monomers.

**Figure 8:**
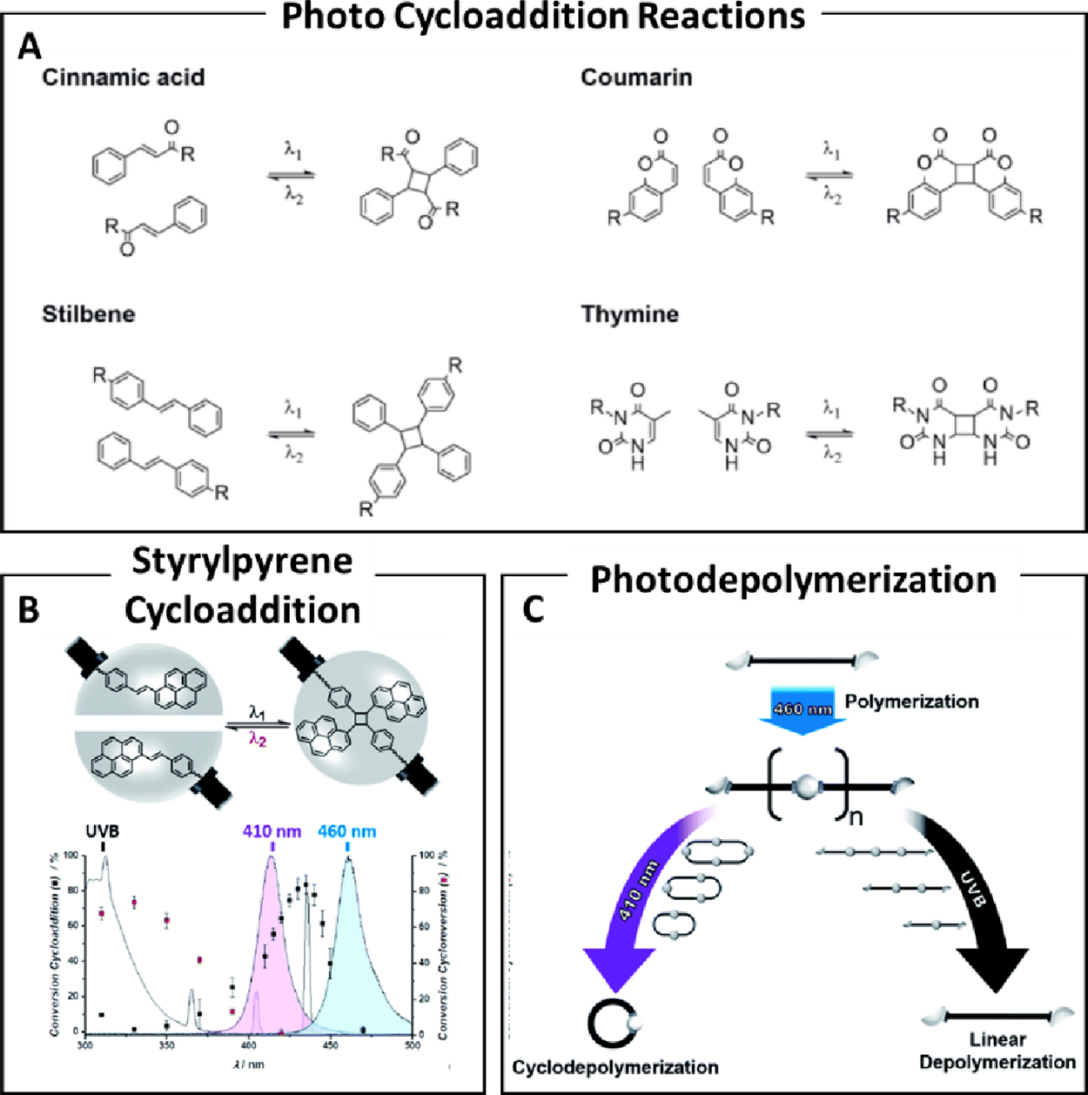
(A) Examples of photoresponsive motifs undergoing reversible [2π + 2π] photocycloaddition. R denotes potential sites for polymer or functional group attachment. (B) Top figure: molecular structure illustrating the reversible [2π + 2π] photocycloaddition of styrylpyrene, employed as a light-responsive binding site in telechelic polymers. Bottom figure: Action plot depicting the wavelength-dependent behavior of the cycloaddition (black markers) and cycloreversion (red markers) reactions of hydroxyl-styrylpyrene, triggered by equal photon doses at varying wavelengths. Overlaid are the normalized emission profiles of the UVB lamp (gray) and LEDs (violet, λ_max = 410 nm; blue, λ_max = 460 nm), showing their respective activation windows. (C) Schematic of polymerization and depolymerization of telechelic polymer L1 with terminal styrylpyrene groups. Blue light induces photopolymerization to form P1, which undergoes cyclodepolymerization under violet light or linear depolymerization under UVB light. Reproduced from ref 67 licensed under CC-BY 4.0 Copyright 2022, CHIMIA, and ref 91 licensed under CC-BY 4.0 Copyright 2020, Royal Society of Chemistry.

Chen and co-workers were among the first to explore the reversible chain extension of coumarins integrated into polymer backbones such as polyesters,^73^ polyethers,^74^ and polyurethanes.^75^ Their investigations demonstrated the wavelength-dependent photoextension and photocleavage using UV–vis spectroscopy and viscosity analysis. However, these studies revealed persistent challenges, including limited reaction yields (typically below 80%) and poor orthogonality—both of which contribute to low molecular weights, resulting in poor material properties and limited degradability.

Additional challenges arise from low selectivity during irradiation, where multiple dimers and alkenes form simultaneously, complicating reaction control and limiting reversibility.^67,76–78^ A major limitation is the photostationary state—an equilibrium between cycloaddition and cycloreversion reactions due to overlapping absorption spectra of reactants and products, which hinders selective reversion by causing simultaneous forward and reverse reactions under the same wavelength. While in solution, differences in diffusion and the kinetics of unimolecular (reversion) versus bimolecular (cycloaddition) reactions can be leveraged to achieve near-orthogonal behavior, the restricted mobility in the solid state prevents effective separation of these photochemical processes, limiting reversion efficiencies to approximately 15%.^79–81^ To overcome these barriers, various strategies have been explored, including structural modifications of the photoresponsive moieties,^82–84^ adjustments in solvent polarity,^85^ and the addition of photosensitizers,^86^ among others—all aimed at enhancing reaction selectivity, improving yields, and increasing reversibility.^65,67^

An alternative approach by Saito and colleagues employed solid-state topochemical polymerization, leveraging preorganized monomer arrangements to achieve controlled, reversible polymerization.^87^ In this approach, crystalline thymine-based monomers were aligned in ordered rows via solvent casting, forming trans–antitype pairs that meet the Schmidt rule’s requirement,^88–90^ which stipulates that the photoresponsive moieties must be within a 4 Å distance to enable cycloaddition. Irradiation at 302 nm produced polymers with *M*_n_ = 1.9 × 10^4^; however, initial attempts at depolymerization using 240 nm light were hindered by photostationary effects, which were resolved by suspending polymer films in acetonitrile for quantitative and selective depolymerization (Table 1, entry 17). Repeated cycles of topochemical photopolymerization and solution-phase depolymerization demonstrated the system’s robust recyclability. Although the polymers exhibited moderate molecular weights and broad dispersity, this method marks a pioneering framework for reversible photopolymerization. To improve sustainability, future efforts could focus on suppressing or replacing the solvent, for example, by optimizing solid-state depolymerization conditions or employing greener solvent alternatives, thereby enhancing the environmental compatibility of the process.

Another example of efficient photorecycling was developed by Barner-Kowollik and co-workers, who demonstrated reversible photopolymerization of high-molecular-weight polyethylene glycol (PEG) chains end-functionalized with styrylpyrene (Figure 8B,C).^91^ Using ″action plots″ that map wavelength-dependent photoreactivity,^92,93^ the team identified optimal wavelengths for cycloaddition (435 nm) and reversion (330 nm) of styrylpyrene.^92^ Based on these findings, PEG-styrylpyrene oligomers were extended under blue LED light to polymers with an unprecedented molecular weight of 60,000 g/mol. Subsequent UVB irradiation enabled complete gated depolymerization within 10 min, achieving full conversion (Table 1, entry 18).

Precisely mapping wavelength-dependent photoreactivity is fundamental for achieving efficient, selective control over photopolymerization and depolymerization, since it enables targeted activation of specific reactions without unwanted side processes—an essential requirement for designing robust, fully photorecyclable materials. Despite these advances in solution-phase photorecycling, translating such efficiency to solid-state systems remains challenging. Limited light penetration in thick materials hinders uniform reversion, necessitating new strategies to achieve complete depolymerization throughout the polymer matrix.

## 4 Mechanochemical Depolymerization

The utilization of mechanical forces to promote chemical reactions has emerged as a highly effective, environmentally friendly alternative to traditional stimuli. In the realm of polymer chemistry, mechanochemistry has been used for triggering reactions within stimuli-responsive polymers, providing precise control of the bond scission events.^94–96^ Polymer mechanochemistry has recently attracted significant attention, particularly for its applications involving mechanophores as failure reporters and mechanical activation for polymer deconstruction.^97–100^

Mechanical force provides access to depolymerization regions of the PES that are otherwise inaccessible via temperature or light. Under an external force, the energy minima and bond breaking points of the PES can shift significantly. For a simple A–B–C model molecule, the bond breaking points for the two valleys can be found along the green lines, and the two Newton Trajectories along the valleys are shown in blue (Figure 9A). Pulling the A–B–C system in opposite directions changes the PES with a concomitant change of the BBPs and minima, leading to stabilization of the transition state and destabilization of the minimum (Figure 9B). For a complete discussion of the model development and other more complex cases, readers are referred to the work by the Quapp group.^101^ Consequently, force is envisioned as an exceptionally effective stimulus for promoting depolymerization. By understanding the PES under the influence of force and applying this knowledge rationally, it is possible to promote depolymerizations through pathways that minimize side reactions and prevent the formation of unwanted byproducts, yielding the desired monomer.

**Figure 9:**
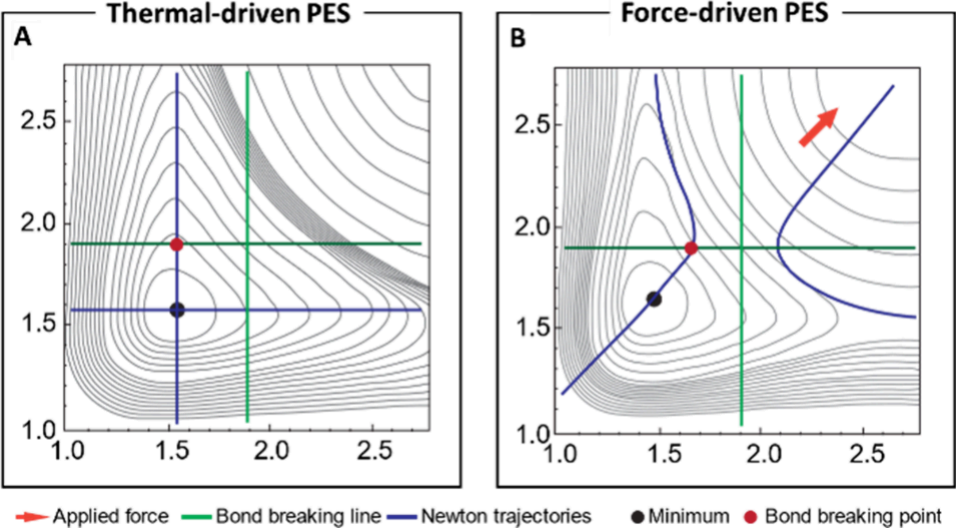
(A) Symmetric 2D-Morse potential for an A–B–C system. (B) Effective 2-D Morse potential for an A–B–C system with *f*_(1,1)_ = 25. The NT to direction *f*_(1,1)_ are shown in blue. Reproduced and adapted with permission from ref 101. Copyright 2018, International Journal of Quantum Chemistry.

However, fully depolymerizing materials into their constituent monomers using forces has been a significant challenge in the field due to the uncontrolled nature of mechanical activation, which often prevents the selective formation of the constituent monomers. Additionally, some reports that explore the use of mechanical forces in the deconstruction of polymers back to their constituent monomers lack critical information, such as monomer recovery yield and purity, making it difficult to assess the feasibility of these methods as alternatives to traditional depolymerization techniques.

### 4.1 Sonication

Sonication-induced elongational flow in linear polymer solutions has been widely employed to activate mechanophores—labile functional groups embedded within polymer chains.^102^ This activation occurs when collapsing cavitation bubbles generate elongational forces, which scale with the chain length of the polymer. Once a critical molecular weight is reached, activation ceases, depending on polymer type and experimental conditions.^103^ Most linear stimuli-responsive polymers degrade into lower-molecular-weight fragments rather than repolymerizable monomers. However, some studies have demonstrated monomer recovery using low-ceiling-temperature polymers, where mechanical activation initiates an unzipping reaction (Figure 10A).

**Figure 10:**
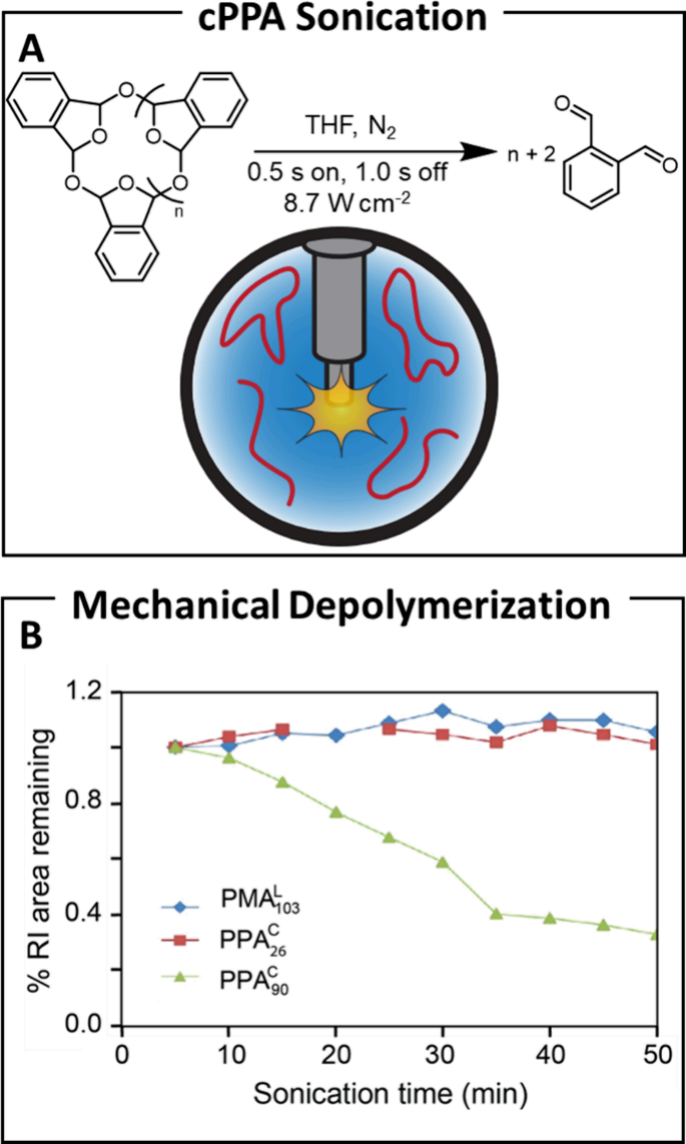
(A) Sonication scheme used for the solution depolymerization of cyclic poly­(*o*-phthalaldehyde). (B) Remaining amount of poly­(*o*-phthalaldehyde) as a function of sonication time. Reproduced with permission from ref 104. Copyright 2014, Nature Chemistry.

The Moore group introduced a mechanochemically degradable polymer, cyclic poly­(*o*-phthalaldehyde) (cPPA^90^) with *M*_w_ = 90 kDa, synthesized via cationic polymerization. When subjected to mechanical activation through pulsed ultrasonication (0.5 s on, 1.0 s off, 8.7 W cm^–2^), chain scission ensues, resulting in the reduction of cyclic polymer content—a response not observed in a lower molecular weight comparator (26 kDa, cPPA^28^) or low molecular weight linear polymethacrylate (PMA^103^) (Figure 10B and Table 1, entry 19).^104^ A mechanistic pathway was proposed in which mechanically driven heterolytic scission, supported by *ab initio* calculations, initiates polymer unzipping from both chain ends, ultimately yielding *o*-phthalaldehyde. The concept was further validated by demonstrating a depolymerization–repolymerization sequence, marking the first demonstration of mechanically induced polymer deconstruction to monomers in solution.

Another example of a polymer unzipping through mechanical stimulation in solution was reported by Kumar and Goodwin. They demonstrated the mechanical depolymerization of poly­(vinyl acetate-*alt-*sulfur dioxide) into sulfur dioxide and vinyl acetate.^105^ Ultrasonication triggered depolymerization, which has shown to proceed even after sonication ceased. After 4 h of ultrasonication, the polymer content was reduced by ∼80%; however, incomplete degradation resulted in persistent oligomers averaging 14 kDa (Table 1, entry 20). This study also demonstrated the potential of ultrasonication as an initiating mechanism for the deconstruction of low ceiling temperature polymers.

### 4.2 Ball Milling

Ball milling is a powerful mechanochemical activation process that harnesses the grinding media collisions to activate polymer structures, inducing cleavage and depolymerization.^106,107^ This little-to-no solvent, energy-efficient approach technique has been successfully applied to deconstruct a variety of commodity polymers, such as polyethylene, polystyrene, and polyvinyl chloride, offering a more sustainable alternative to traditional polymer waste management. However, achieving selective depolymerization remains a challenge.^108^ Despite its promise, the ability of ball milling to fully break down polymers into monomers suitable for repolymerization remains largely underexplored with only a limited number of studies successfully demonstrating monomer recovery for reuse (Figure 11A).

**Figure 11:**
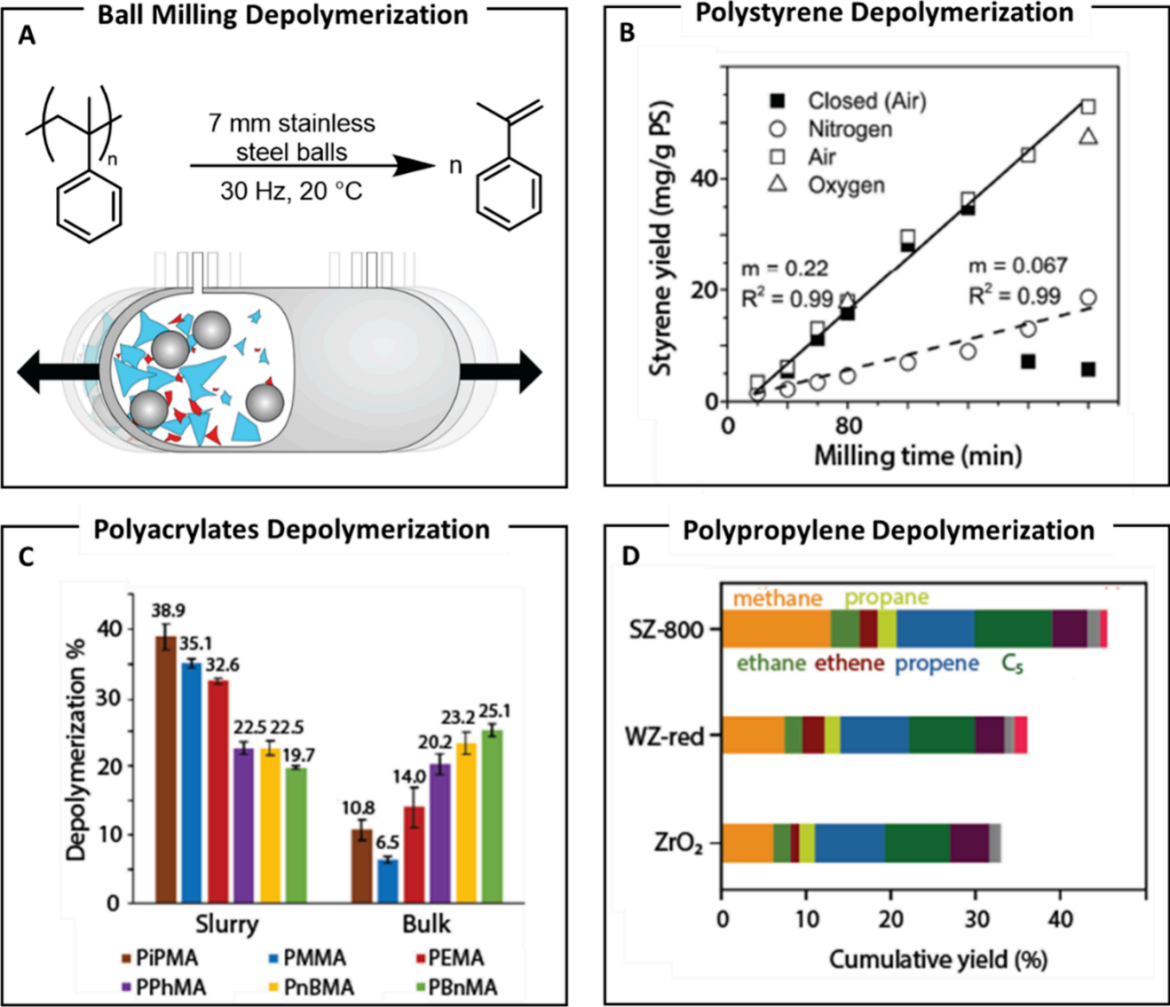
(A) Ball milling scheme for the deconstruction of poly­(α-methyl styrene). (B) Styrene production via ball milling over time under four different ball-milling conditions. Reproduced from ref 110. Copyright 2023 American Chemical Society. (C) Comparison of polyacrylate depolymerization based on acrylate side chains and ball milling conditions. (D) Polypropylene depolymerization using different spheres. Reproduced from ref 98. Copyright 2024 American Chemical Society.

#### 4.2.1 Ball Milling of Polyolefins

Balema et al. employed ball milling to depolymerize PS, yielding only 7.0% styrene after 12 h. While the authors attributed depolymerization to the mechanochemically generated radicals, the reasons for the low yield were left unexplained (Table 1, entry 21).^109^ More recently, the Sievers group improved styrene selectivity (up to 80%) by introducing a continuous gas stream to remove volatile monomers.^110^ However, the overall yield remained low, reaching only 5.5% after 4 h (Figure 11B and Table 1, entry 22). The authors posit that both PS chain scission and depolymerization initiate with homolytic C–C bond rupture, leading to different reaction pathways depending on oxygen availability. Milling with SiN balls was less effective than with Fe or W, and the introduction of air resulted in enhanced depolymerization. The proposed mechanism involves carbon-centered radicals reacting with oxygen to form peroxides, which interact with metal surfaces to catalyze further depolymerization.

Another report by the Choi group also investigated the mechanically induced depolymerization of PAMS,^111^ resulting in significantly higher yield of 55% α-methyl styrene with 80% selectivity under similar ball-milling conditions (Table 1, entry 23). The depolymerization was attributed to the low ceiling temperature of PAMS (61 °C), with evidence from the dependence on the parent polymer’s molecular weight, CoGEF simulations, and radical trapping experiments indicated that mechanochemical chain scission initiates depolymerization through a radical mechanism, even below the ceiling temperature. It was also found that oxygen has minimal impact on the extent of depolymerization, contrasting with Balema et al.’s findings where PS showed negligible depolymerization in an argon atmosphere, but significant depolymerization in air. This suggests a distinct depolymerization pathway for PMS compared to PS, possibly due to PMS’s low ceiling temperature.^110^

In a recent study, the Choi group, in collaboration with the Peterson group, expanded their work on PAMS by investigating the ball milling of PMAs.^112^ The authors discussed that although ball milling has been used to depolymerize polymethyl methacrylate to methyl methacrylate (MMA), the initial reaction yield was only 4%. In their study, the authors significantly increased the MMA yield to 41% by employing slurry conditions with *t*-BuOH and gentle heating at 43 °C (Table 1, entry 24). Interestingly, as with PS depolymerization, oxygen appears to influence the unzipping of PMAs. Electron spin resonance experiments indicated that PMMA reacts with oxygen to form peroxide radicals,^113^ similar to those proposed in PS mechanochemical depolymerization, suggesting a comparable mechanism for PMMA. However, the authors emphasize that further studies are needed to fully clarify this depolymerization mechanism. Depolymerization of PMAs was more efficient in slurry than in bulk, and recovery under slurry conditions was inversely correlated with substituent steric size, whereas in bulk, it was directly correlated (Figure 11C). Large-scale PMMA depolymerization yielded a low amount of MMA, but continuous monomer removal improved yield from 16.8 to 26.4%, highlighting its potential to enhance efficiency with minimal impact on chain scission. Ball milling of PMAs further supports its potential for recycling vinyl-based polymers.

The expansion of ball milling in the depolymerization of more challenging polyolefins was conducted by the Vollmer group, which recently reported a mechano-catalytic strategy for converting PP into small hydrocarbons.^114^ By optimizing milling parameters—such as shaking frequency and filling degree—they achieved 46% conversion to C_1–10_ hydrocarbons and H_2_ within 1 h using surface-activated sulfated grinding spheres. However, only 10% reverted into propane, underscoring the need for improved selectivity (Figure 11D and Table 1, entry 25). In this process, the catalyst likely stabilizes mechano-radicals, forming reversible adducts that extend radical lifetimes and favor selective decomposition. Furthermore, sulfated zirconia effectively catalyzed PP, PE, PS, and industrial waste degradation, increasing monomer recovery for PE and PS. This innovative approach highlights a viable mechanochemical pathway for propylene deconstruction and its differentiation from thermochemical deconstruction.

Sievers’ group conducted a comprehensive thermodynamic study on the mechanochemical depolymerization of commodity polymers via ball milling.^115^ They proposed that depolymerization is a surface-driven process, facilitated by particle fracture and microscopic friction. Their theoretical framework estimated depropagation equilibrium constants for PS, PP, and PE, assuming kinetic energy from grinding converts into thermal energy. Results showed depolymerization follows the trend PS > PP > PE, aligning with their ceiling temperatures. The study also found that styrene repolymerizes more readily than PP and PE due to its difficulty in being removed from the system. Depolymerization was determined to be more limited by the energy required for depropagation than by macroradical formation. Thus, creating energy-rich domains—such as larger, hotter, and longer-lasting hotspots—is key to improving mechanochemical recycling. To enhance efficiency under milder conditions, the authors suggested coupling depolymerization with hydrogenation or oxidation. Additionally, efficient monomer removal could aid thermodynamically constrained depolymerization, though each monomer’s physical properties must be considered.

#### 4.2.2 Ball Milling of PET

Sievers and Štrukil independently demonstrated that combining ball milling with sodium hydroxide enables solid-state alkaline hydrolysis of PET’s ester linkages. Štrukil achieved up to 96% PET conversion and 93% terephthalic acid (TPA) yield in 1 h, which was further optimized by modifying the base, ball material, and size and adding solvent, enabling 98% PET conversion and 97% TPA yield (Table 1, entry 26). The reaction remained selective for ester hydrolysis even when PET was mixed with polyolefins like PS and PP. Finally, the author scaled up the reaction from 0.5 to 4 g, achieving full PET conversion and TPA recovery after 3 h of milling.^116^

Sievers’ group later conducted a detailed mechanistic study, achieving 100% PET conversion in just 17 min with >90% TPA selectivity (Figure 12 and Table 1, entry 27). They attributed the higher efficiency compared to Štrukil’s study to the larger ball size (20 vs 12 mm). By tracking PET molecular weight and polydispersity, they observed extended hydrolysis in high-MW PET. Further experiments examined the impact of milling parameters, showing that TPA yield increases with frequency, ball mass, and vessel temperature. Complete depolymerization was achieved in 20 min when the ball-to-powder ratio (BPR) was ≥20, while for BPR <10, TPA production dropped below 20%.^117^ They proposed a two-step depolymerization: a slow solid-phase reaction followed by a higher-yield waxy phase but with lower selectivity. Milling increased monomer yield but left some intact chains, supporting a shrinking core model. Unlike surface-area-dependent models, mechanochemical hydrolysis relies on particle deformation and fracture, requiring a new model to capture these dynamics.

**Figure 12:**
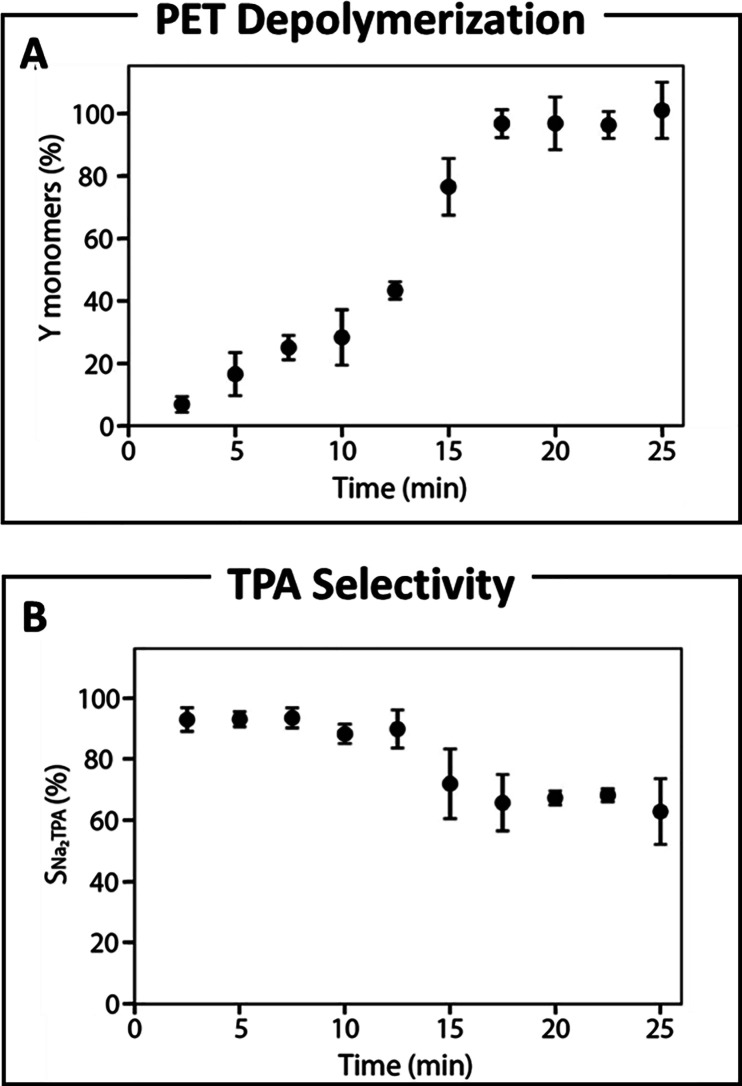
(A) Depolymerization of polyethylene terephthalate over time using ball milling with equimolar sodium hydroxide. (B) Selectivity toward the production of terephthalic acid. Reproduced from ref 117. Copyright 2022 American Chemical Society.

Mechanical forces have been shown to break down polymers into their original monomers in some commodity polymers and even in more specialized examples. Despite these advances, challenges remain regarding depolymerization yield and selective conversion to constituent monomers. Sonication has been used to disassemble polymers in solution without additional reagents, though it is constrained by polymer molecular weight, which can lead to oligomeric side products. Furthermore, this method requires specific conditions such as dilute solutions, low temperatures, and an inert atmosphere. Ball milling, a commonly used method for breaking down commodity polymers, often relies on additives to aid deconstruction but suffers from low recovery yields and limited selectivity, reducing its practical applicability. The impact of mechanical forces on the PES has not been broadly explored for depolymerization and force as a stimulus is often viewed merely as an extension of thermal stimulation. We propose that by better understanding the force-modified PES and applying this knowledge strategically, we can harness mechanical forces to drive depolymerization along pathways that minimize side reactions, reduce byproduct formation, and improve monomer recovery. Consequently, novel approaches for applying mechanical stimulation as well as novel recycling units should be considered to improve the use of mechanical forces in the deconstruction of polymers back to their constituent monomers.

**Table 1:** Level of intervention in the deconstruction of different polymers using heat, light, and force as stimuli

entry	system	mechanism/process	reaction conditions	concentration (mg/mL)	solvent/additives	recovery yield (%)	purity/selectivity	ref.
	*2.0 Bulk, thermal depolymerization of commodity materials*							
1	PE	cold plasma	500–700 °C	bulk	zeloites	22–24%	N/A	^29^
2	PP	traditional pyrolysis	pyrolysis (N_2_) at 20 °C/min	bulk	none	10%	N/A	^30^
3	PP	induction-coupled plasma	2700–7700 °C at 10^6^ °C/s, argon	bulk	none	78%	94%	^33^
4	PP	electrified spatiotemporal heating	*T*_max_ = 600 °C, 0.11 s	bulk	none	36% (locally)	N/A	^34^
5	PET	electrified spatiotemporal heating	*T*_max_ = 1050 °C, 0.11 s	bulk	none	43% (locally)	N/A	^34^
6	PMA	endgroup alkenes or RAFT chain-transfer agents, thermolysis	220 °C, N_2_, 4 h	bulk	none	84%	high-purity NMR	^36^
7	PMMA *copolymer*	pendent group activation (phthalimide decomposition)	250 °C, vacuum, 2.5 h	bulk	none	81%	high-purity NMR	^37^
8	PMMA *copolymer*	backbone activation (alpha methyl styrene-type monomers)	180 °C, 6–8 h	bulk	none	78%	high-purity NMR	^39^
9	PMMA *block copolymer*	pendent group activation (phthalimide decomposition)	290 °C	bulk	none	N/A	high-purity NMR	^40^
10	PAMS *block copolymer*	pendent group activation (phthalimide decomposition)	220 °C	bulk	none	70%	high-purity NMR	^40^
11	biobased poly(methylene butyrolactones)	backbone activation	370–390 °C, at 100 mTorr, 1 h	bulk	none	83–99.8%	high-purity NMR	^41^
	*3.0 Photodriven depolymerization*							
12	poly(olefin sulfone)	pendent group activation	UV light (254nm, 0.85–1.4 kJ cm^–2^), 150 °C	bulk	none	50%	mass with GPC, no NMR	^56^
13	PMMA	pendent group activation RAFT	green light (515 nm, 0.5 mW cm^–2^), UV (365 nm, 0.7 mW cm^–2^), 100 °C, 1 h	5	1,4-dioxane	87%	N/A	^61^
14	PMMA	pendent group activation RAFT	green light (510 nm, 2.31 mW cm^–2^), 100 °C, 8 h	0.5	1,4-dioxane/eosin Y (100 ppm)	80%	N/A	^62^
15	PBzMA	pendent group activation ATRP	blue LED (460 nm, 7.26 mW cm^–2^), 100 °C, 8 h	9	anisole/FeCl_2_ of FeCl_3_ (0.05 eq relative to polymer unit)	90%	N/A	^63^
16	PAMS, PPA, PMMA, PS, PLA	uncontrolled deconstruction	white LED (6000 K color temperature), 20 °C, 30 min	bulk	10–20 wt% CQDs + ZnO (0.5 eq) or BaO (0.07 eq)	85%	N/A	^64^
17	alkyl chains	uncontrolled deconstruction	UV light (240 nm, 0.85–1.4 kJ cm^–2^), 20 °C, slurry	N/A	ACN	100% after 17.5 min	100%	^87^
18	PEG	uncontrolled deconstruction	UV light (330 nm), 20 °C, 10 min	0.05	THF	100% after 50 min	100%	^91^
	*4.0 Mechanochemical depolymerization*							
19	cPPA	sonication	–15 °C under Ar	1	THF	62% after 50 min	high-purity NMR	^104^
20	poly(olefin sulfone)	sonication	–10 °C under Ar	10	acetone	90% after 1 h	N/A	^105^
21	PS	ball milling	air flux	bulk	none	7% after 12 h	NA	^109^
22	PS	ball milling	air flux	bulk	none	5.5% after 4 h	80%	^110^
23	PAMS	ball milling	standard	bulk	none	55% after 8 min	99%	^111^
24	PMMA	ball milling	44 °C, slurry	300	^t^BuOH	41.1% after 20 min	N/A	^112^
25	PP	ball milling	40 °C, N_2_ flux	bulk	none	46% after 1 h	10%	^114^
26	PET	ball milling	sealed jar	bulk	NaOH (29.6%)	96% after 1 h	93%	^116^
27	PET	ball milling	sealed jar	bulk	NaOH (29.6%)	100% after 17.5 min	91%	^117^

## 5 A Forward-Looking Perspective on Stimuli-Driven Depolymerization

Depolymerization has emerged as one of the most promising strategies to address the challenge of plastic waste by enabling monomer-level recycling. As this Perspective has shown, stimuli-driven depolymerization—activated by heat, light, or mechanical force—has led to significant advances in our ability to recover valuable building blocks from polymeric materials. Yet despite these technical achievements, the field remains fragmented, and the path to scalable, circular implementation is far from resolved.

Thermal depolymerization remains the most industrially mature approach and benefits from infrastructure already in place. However, its high energy requirements and low product selectivity continue to limit its sustainability. Recent advances such as rapid spatiotemporal heating and the use of weak-link chemistries demonstrate that both kinetic and thermodynamic barriers can be addressed through process and materials design. Still, these approaches often apply only to model systems or require feedstock purity not achievable in real-world waste streams.

Photochemical strategies offer precision and operate under milder conditions, avoiding many of the side reactions inherent to thermolysis. Tailored light activation has enabled selective bond scission in RAFT and ATRP polymers, and photothermal hybrid systems now demonstrate monomer recovery from bulk materials. However, limited light penetration, reliance on dilute solution conditions, and specialized chemistries still restrict broader implementation. Solid-state photodepolymerization remains a major technical hurdle—one that may require new material architectures or light-guiding systems to overcome.

Mechanical activation has gained attention for its potential as a little-to-no solvent, low-energy method, particularly via ball milling and sonication. Still, most reports yield oligomeric products or exhibit poor selectivity. That said, the integration of mechanophores and the development of hybrid photomechanical or thermo-mechanical pathways point to a future where force itself becomes a useful and tunable stimulus. Mechanochemistry has also shown unexpected promise in enabling depolymerization in heterogeneous systems, which may prove essential in processing real-world waste.

It becomes evident that optimizing a single stimulus alone is insufficient. Each method offers a specific benefit—energy efficiency, selectivity, accessibility—but also a tradeoff. As such, the future of depolymerization lies in the deliberate design of multimodal strategies: systems that combine thermal, photochemical, and mechanical inputs to enable efficient, selective, and responsive breakdown under realistic conditions. Beyond recycling, these same design principles open the door to entirely new applications: Depolymerization has been explored as a platform for stimuli-gated information encryption and data storage,^118^ transient materials for biomedical uses,^119^ and responsive systems for soft robotics.^120^ Such examples highlight that depolymerization is not only a route to circularity but also a versatile chemical strategy with potential to impact fields well outside sustainability. To meaningfully compare emerging multimodal strategies, standardized reporting of key performance metrics—such as reaction conditions, yield, energy input, monomer purity, selectivity, and polymerization degree—must become the norm. As highlighted in Table 1, these variables are frequently underreported, particularly purity and selectivity, which are essential for assessing the quality and reusability of recovered monomers. Equally important is the downstream step: monomer reintegration. While many studies focus on recovery, few assess whether the regenerated monomers can be repolymerized effectively or match the performance of virgin materials. Standardized protocols for evaluating monomer stability, reactivity, and material properties are critical to ensure that depolymerization contributes to true circularity, rather than becoming another form of downcycling. Without these shared benchmarks, the field risks fragmenting into isolated successes with limited scalability.

## 6 Conclusion: Toward a Functional Depolymerization Ecosystem

The future of depolymerization is not defined by a single breakthrough or stimulus, but by the integration of chemistry, materials science, and process engineering to create holistic solutions. This requires a paradigm shift: away from isolated stimulus optimization and toward designing systems where polymers are made to be unmade—selectively, efficiently, and repeatedly. Just as thermoplastics once revolutionized materials science, depolymerizable plastics—responsive to force, light, and heat—could redefine material reuse at the molecular level.

To get there, collaborative effort is essential. Chemists, physicists, engineers, and industrial partners must align their work not only with academic goals but with infrastructure, economic, and regulatory realities. Depolymerization must graduate from proof of concept to process platform. The adoption of standardized metrics—such as those summarized in Table 1—will be essential to benchmark performance, facilitate comparison across systems, and support the emergence of a truly functional depolymerization platform. If successful, then these advances will not only reduce waste—they will transform the very relationship between synthesis, use, and reuse, enabling a truly circular polymer economy. Table 1 provides a curated overview of key parameters that should be consistently reported in future depolymerization studies. These include not only process variables (e.g., mechanism, additives, and conditions) and recovery yield but also monomer purity, selectivity, and the outcome of any repolymerization attempts. Establishing such benchmarks is critical to assessing the feasibility of different depolymerization strategies and to distinguishing between one-time chemical degradation and truly circular recycling systems. Broad adoption of these reporting standards will ensure that new developments are not just technically innovative but also translatable, comparable, and industrially meaningful. As these technologies mature, questions of economic feasibility, environmental impact, and regulatory alignment will become increasingly relevant and warrant dedicated attention in future work.
